# Quantum Geometric Engineering of Dual Hall Effects in 2D Antiferromagnetic Bilayers via Interlayer Magnetic Coupling

**DOI:** 10.1002/advs.202505860

**Published:** 2025-05-28

**Authors:** Zhenning Sun, Tao Wang, Hao Jin, Xinru Li, Yadong Wei, Jian Wang

**Affiliations:** ^1^ College of Physics and Optoelectronic Engineering Shenzhen University Shenzhen 518060 P. R. China; ^2^ School of Physics State Key Laboratory of Crystal Materials Shandong University Shandanan Street 27 Jinan 250100 P. R. China; ^3^ Department of Physics The University of Hong Kong Pokfulam Road Hong Kong 999077 P. R. China

**Keywords:** altermagnetism, anomalous Hall effect, interlayer magnetic coupling, nonlinear hall effect, quantum geometry

## Abstract

The interplay between quantum geometry and magnetic order offers a novel strategy for designing next‐generation nanodevices. Here, it is demonstrated that interlayer magnetic coupling in two‐dimensional (2D) CoPSe_3_ bilayers enables precise control over quantum geometric mechanisms, unlocking dual intrinsic Hall effects. The first‐principles calculations reveal that the altermagnetic (AM) phase exhibits a giant anisotropic anomalous Hall effect (AHE) (σ_*xy*_ ≈46 S cm^−1^) driven by Berry curvature localized at generic *k*‐points, while the PT‐symmetric antiferromagnetic (AFM) phase hosts an intrinsic second‐order nonlinear anomalous Hall effect (NAHE) (χ_*xyy*_ ≈ 160 µS V^−1^) originating from quantum metric accumulation at high‐symmetry *k*‐points. By tuning interlayer magnetic couplings, reversible switching between these phases is achieved, leveraging their distinct band structures and symmetry constraints. The Néel‐vector‐dependent AHE in the AM phase and the symmetry‐protected NAHE in the AFM phase highlight quantum geometry as a versatile tool for manipulating transport properties. This work establishes 2D antiferromagnets as a promising platform for multifunctional device architectures, bridging linear and nonlinear magnetoelectric responses through tailored quantum geometric engineering.

## Graphical abstract

Precise tuning of interlayer magnetic coupling in two‐dimensional bilayers enables control over altermagnetic and PT‐symmetric antiferromagnetic phases. This dual‐phase platform yields two intrinsic Hall responses: a Berry curvature–driven linear anomalous Hall effect and a quantum metric–induced second‐order nonlinear anomalous Hall effect, highlighting the interplay between magnetic order and quantum geometry in transport phenomena.

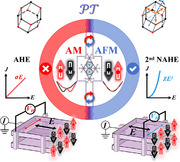

## 1 Introduction

Quantum geometric tensor, which characterizes the geometric properties of electronic wavefunctions in momentum space, plays a fundamental role in determining the transport properties of materials, particularly the Hall effects.^[^ ^1^, ^2^, ^3^, ^4^, ^5^ ^]^ This quantum geometric tensor has both real and imaginary components,^[^ ^6^, ^7^, ^8^, ^9^, ^10^, ^11^, ^12^, ^13^, ^14^ ^]^ each contributing uniquely to the Hall response. The imaginary part, known as the Berry curvature, is central to the intrinsic anomalous Hall effect (AHE), a phenomenon that is independent of the relaxation time τ and is typically observed in ferromagnetic materials (FMs), where time‐reversal symmetry (T) is broken. However, antiferromagnetic materials (AFMs), characterized by zero net magnetization and symmetric magnetic configurations, have long been overlooked in AHE studies.^[^ ^15^, ^16^ ^]^ Despite this, AFMs offer unique advantages, such as ultrafast dynamics, robustness against external perturbations, and distinctive magnetotransport properties. Recent work has further shown that the Néel vector can control symmetry‐allowed nonlinear responses, including hidden valley‐polarized photoconductivity in AFM systems.^[^ ^17^ ^]^ These results highlight the broader potential of AFMs as versatile platforms, making them highly attractive for future spintronic technologies.^[^ ^18^, ^19^, ^20^, ^21^ ^]^

Recently, a new class of AFMs, known as altermagnets (AMs),^[^ ^22^, ^23^, ^24^, ^25^, ^26^ ^]^ has attracted considerable attention due to their unconventional properties. These materials exhibit the alternating nature of the spin polarization in momentum space and nonrelativistic spin splitting occurring at generic *k*‐points. Despite having zero net magnetization, AMs can exhibit pronounced anomalous Hall signals under specific magnetic symmetries, challenging the traditional understanding of the intrinsic AHE in AFMs.^[^ ^27^, ^28^ ^]^ Experimental studies on materials such as RuO_2_ and MnTe,^[^ ^22^, ^29^, ^30^, ^31^ ^]^ which feature collinear compensated magnetic configurations, have provided direct evidence linking the observed AHE to the altermagnetic (AM) phase. The key distinction between AMs and conventional AFMs arises from their symmetry constraints. In typical AFMs, PT‐symmetry enforces spin degeneracy across the Brillouin zone even with spin‐orbit coupling (SOC). This symmetry prohibits the formation of finite Berry curvature required for an intrinsic AHE. By contrast, AMs can lift band degeneracy at generic *k*‐points in the absence of SOC.^[^ ^32^ ^]^ Consequently, AMs with certain magnetic symmetries can exhibit finite Berry curvature and yield a sizable intrinsic Hall response, offering new insights into the AHE in AFM systems.^[^ ^33^, ^34^, ^35^ ^]^

While the Berry curvature is central to the linear intrinsic AHE, the real part of quantum geometry, known as the quantum metric,^[^ ^7^, ^8^, ^10^, ^36^, ^37^, ^38^ ^]^ plays an equally important role in transport phenomena,^[^ ^39^, ^40^ ^]^ particularly in the intrinsic nonlinear anomalous Hall effect (NAHE). In PT‐AFM systems, contributions from the Berry curvature and its dipole, which typically drive the intrinsic linear and extrinsic second‐order nonlinear responses,^[^ ^3^, ^36^, ^41^ ^]^ are suppressed due to symmetry constraints. Nevertheless, the quantum metric, allowed by these symmetries, becomes the dominant factor in generating an intrinsic second‐order nonlinear response.^[^ ^37^ ^]^ This τ‐independent NAHE results in a more complex and richer Hall behavior, as observed in PT‐symmetric AFM materials like Mn₂Au and CuMnAs.^[^ ^42^, ^43^, ^44^, ^45^, ^46^ ^]^ These findings highlight the crucial role of the quantum metric, demonstrating its significant impact on the Hall response, particularly in systems where the Berry curvature is excluded by symmetry.

Although significant progress has been made, few studies have managed to investigate these intrinsic Hall responses driven by different mechanisms within the same system. In addition, the intricate interplay between the real and imaginary parts of quantum geometry in shaping Hall responses in AFM systems remains poorly understood. In this work, we bridge this gap by demonstrating that careful manipulation of the interlayer magnetic coupling in a two‐dimensional (2D) AFM bilayer can switch between AM and PT‐symmetric AFM phases. Using CoPSe_3_ bilayers as a model system, we show that this transition enables us to realize two distinct Hall effects simultaneously: the intrinsic linear AHE in the AM phase, governed by the Berry curvature from generic *k*‐points, and the intrinsic second‐order NAHE in the PT‐symmetric AFM phase, driven by the quantum metric from high‐symmetry *k*‐points. Our work not only sheds light on the complex relationship between intrinsic linear and nonlinear Hall responses, but also demonstrates the potential of quantum geometry—particularly in AFMs, which have traditionally been overlooked in spintronics—to be flexibly tuned for novel device applications.

## 2 Computational Details

First‐principles calculations employing the projector augmented wave (PAW) method are performed by the density functional theory (DFT) software Vienna ab initio simulation package (VASP).^[^ ^47^, ^48^ ^]^ The exchange‐correlation function is approximated by the generalized gradient approximation (GGA) within the Perdew–Burke–Ernzerhof (PBE) form.^[^ ^49^, ^50^ ^]^ The criteria of energy and force are set to 10^−6^ eV and 10^−2^ eV Å^−1^, respectively, with a plane wave cutoff energy of 500 eV for the guarantee of convergence. The *k*‐point grid is set to 12 × 12 × 1. Convergence tests for both the cutoff energy and *k*‐point grid are provided in Section SII (Supporting Information), which demonstrate that these parameters achieve a reliable balance between computational accuracy and efficiency. An effective Hubbard *U*_eff_ is applied to the Co*‐d* orbitals by the GGA + *U* (*U* = 4.0 eV) method to include the strong Coulomb interaction on the 3*d* orbitals.^[^ ^51^, ^52^ ^]^ A vacuum layer of 20 Å is added to eliminate uncorrelated interlayer interaction in the periodic calculations. The semi‐empirical dispersion correction (DFT‐D3) method is used to capture nonbonding van der Waals (vdW) interactions.^[^ ^53^, ^54^ ^]^ To evaluate the thermodynamic stability, we perform ab initio molecular dynamics (AIMD) simulation at 300 K. Magnetic exchange parameters are achieved through the four‐state mapping analysis.^[^ ^55^ ^]^ To ensure the reliability of these magnetic exchange parameters, we perform a series of convergence tests by varying key computational parameters, including the supercell size, plane‐wave cutoff energy, and *k*‐point sampling. Additional simulation details are provided in Section SII (Supporting Information). The tight‐binding Hamiltonian that considers Co‐3*d*, P‐3*p*, and Se‐4*p* orbitals is constructed through the maximally localized Wannier functions (MLWFs) generated by the *wannier*90 code.^[^ ^56^ ^]^

Berry curvature Ω and Berry connection polarizability (BCP) G are intrinsically related to the experimentally detected quantity (current density *j*) associated with the AHE and the second‐order NAHE. The anomalous Hall conductivity (AHC) $\sigma_{\alpha \beta }^{\mathrm{AHE}}$ and second‐order intrinsic nonlinear anomalous Hall conductivity (NAHC) tensor χ_*αβγ*_ are defined as

(1) $$\begin{array}{c} \left\{\begin{array}{c} j_{\alpha }^{\mathrm{AHE}\;}=\sigma_{\alpha \beta }^{\mathrm{AHE}}E_{\beta }\\ j_{\alpha }^{\mathrm{NAHE}\;}=\chi_{\alpha \beta \gamma }E_{\beta }E_{\gamma } \end{array}\right. \end{array}$$

here, *α*, *β*, and *γ* are Cartesian coordinates, and *E* is the component of the applied electric field. The AHC is studied using the Kubo formula,^[^ ^1^, ^2^, ^57^ ^]^ which is derived by integrating the Berry curvature across the entire Brillouin zone as

(2) $$\begin{array}{c} \sigma_{\alpha \beta }^{\mathrm{AHE}}=-\frac{e^{2}}{\hbar }\int_{\mathrm{BZ}}\;\frac{dk}{{\left(2\pi \right)}^{2}}\sum_{n}\;f_{kn}\Omega_{\alpha \beta }^{n}\left(k\right) \end{array}$$

*f_kn_* is the Fermi–Dirac distribution function. $\Omega_{\alpha \beta }^{n}\left(k\right)$ is the band‐projected Berry curvature, which is expressed as

(3) Ωαβnk=−2Im∑m≠nnv^αmmv^βnεkn−εkm2

here, |*n*〉 denotes the eigenstates in band *n* with eigenvalues $\varepsilon_{k}^{n}$ and momentum *k*. $\hat{v}$ is the velocity operator. The intrinsic second‐order NAHC is given by^[^ ^37^, ^45^, ^58^, ^59^ ^]^

(4) $$\begin{array}{c} \chi_{\alpha \beta \gamma }=-\int_{\mathrm{BZ}}\;\;\frac{dk}{{\left(2\pi \right)}^{2}}\sum_{n}\;\;f_{kn}\left(\partial_{\alpha }G_{\beta \gamma }^{n}\left(k\right)-\partial_{\beta }G_{\alpha \gamma }^{n}\left(k\right)\right) \end{array}$$

where G^*n*^ is related to the real part of local quantum geometry, known as the BCP tensor. The band‐projected BCP tensor is defined as

(5) Gαβnk=2Re∑m≠nAαnmkAβnmkεkn−εkm

here, $A_{\alpha }^{nm}=\left\langle n|i\partial_{k_{\alpha }}|m\right\rangle$ is the interband Berry connection. In this work, to deal with the rapid variation of the Berry curvature and BCP, a dense *k*‐mesh with 600 × 600 × 1 is employed for the Brillouin zone integration. Moreover, we also apply a logarithmic transformation to the results to promote visualization^[^ ^24^, ^60^ ^]^:

(6) $$\begin{array}{c} \log\left(X\right)=\left\{\begin{array}{c} sgn\left(X\right)lg\left(\left|X\right|\right),\;\left|X\right|>10\\ \;\;\;\;X/10,\;\left|X\right|\leq 10 \end{array}\right. \end{array}$$

here, sgn(X) is the signum function return back to the positive or negative sign of the original X (X=Ω or G).

## 3 Results and Discussion

Symmetry operations have distinct effects on the energy dispersion *E*(*k*, σ). For example, spatial inversion (P) reverses the wave vector (k→−k) without affecting spin, while T reverses both the wave vector and spin (k,σ→−k,−σ). PT‐symmetry enforces spin degeneracy, preventing spin splitting across the entire Brillouin zone. Breaking PT‐symmetry is thus essential to induce spin splitting, which is a prerequisite for magnetoelectronic responses such as AHE. However, achieving spin splitting in AFM systems is challenging due to their inherent symmetry constraints. Notably, in 2D magnetic materials, interlayer magnetic coupling offers a route to manipulate symmetry properties and break PT‐symmetry.

**Figure 1:**
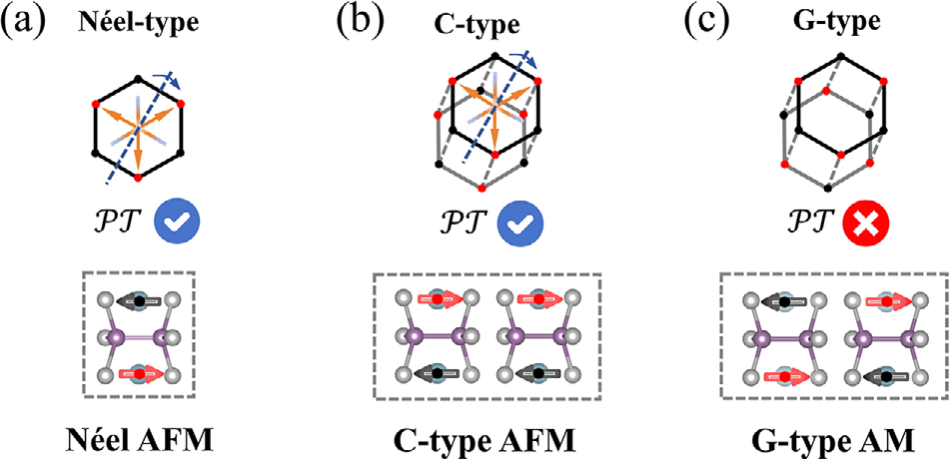
Crystal structures and magnetic configurations of the 2D CoPSe_3_ system. a) The Néel‐type AFM monolayer, b) the C‐type AFM bilayer, and c) the G‐type AM bilayer. Red and black points/arrows represent the magnetic moments of the magnetic ions.

In **Figure** **1**, we take the CoPSe_3_ system as an example and illustrate how interlayer magnetic coupling can manipulate symmetry and break PT‐symmetry. The CoPSe_3_ monolayer features a hexagonal crystal structure with a *D*_3*d*_ point group in its nonmagnetic state. Each Co ion is situated within a distorted CoSe_6_ octahedron, coordinated by six Se ions, with neighboring octahedra sharing edges (see Figure S1, Supporting Information). As shown in **Figure** 1a, the Néel‐type AFM ground state of the CoPSe_3_ monolayer retains PT‐symmetry. Consequently, the CoPSe_3_ monolayer displays zero net magnetic moment and spin degeneracy throughout the Brillouin zone.

In the AA‐stacked bilayer (**Figure** 1b,c), two magnetic configurations, namely C‐type and G‐type, are considered. The C‐type configuration is characterized by AFM intralayer coupling and FM interlayer coupling. This arrangement preserves PT‐symmetry and thus prohibits spin splitting. On the contrary, in the G‐type configuration, both intralayer and interlayer couplings are AFM, leading to the breaking of PT‐symmetry. This symmetry breaking introduces spin splitting in the energy bands and gives rise to an AM phase. First‐principles calculations further confirm that the G‐type AM configuration is energetically more stable than the C‐type AFM configuration in CoPSe_3_​ bilayers.

**Figure 2:**
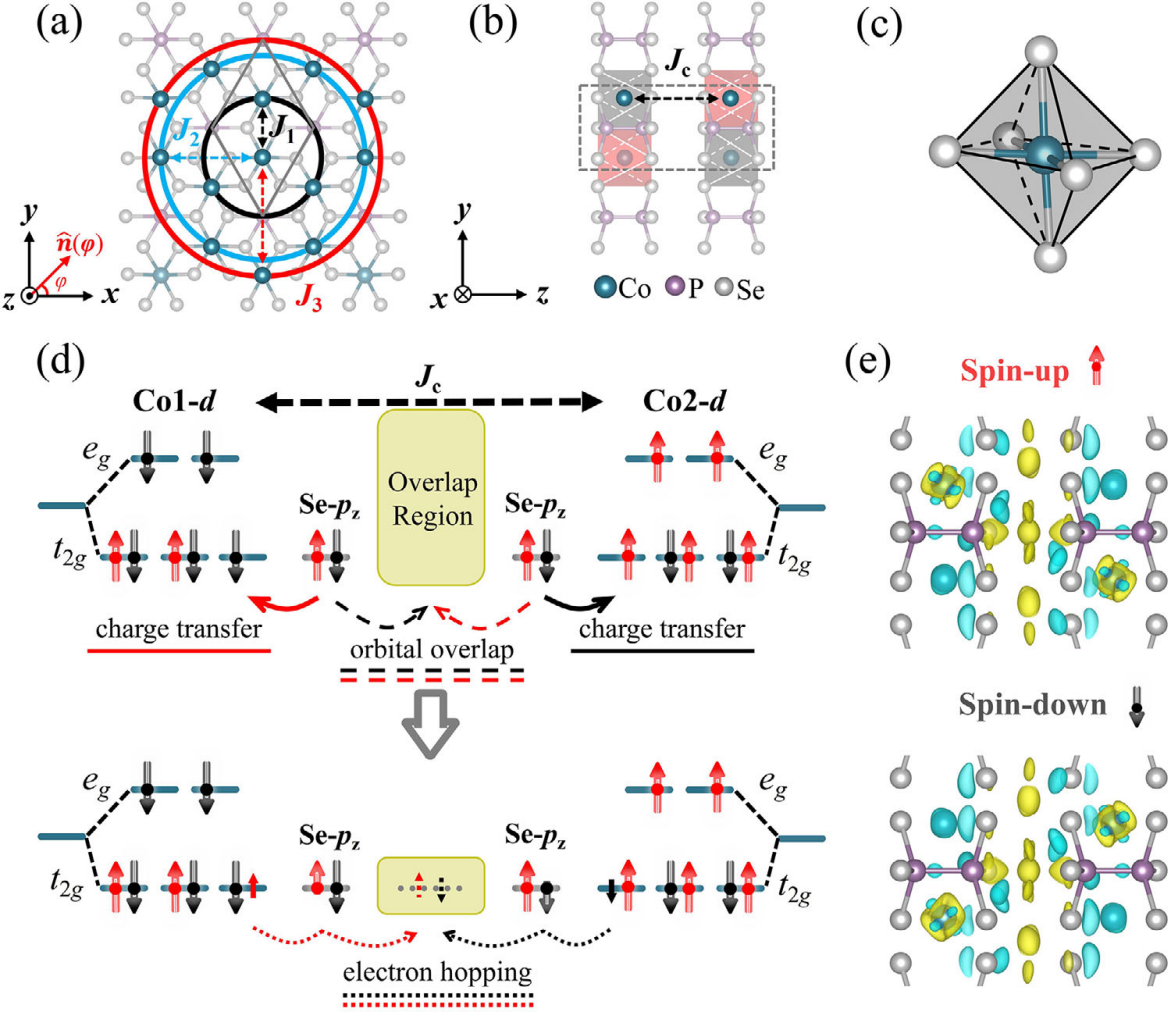
a) Top view of CoPSe_3_ bilayer, with the primitive cell outlined by a solid gray diamond. The nearest, second‐nearest, and third‐nearest neighbors of the central Co atom are marked by black, blue, and red circles, respectively. The corresponding intralayer magnetic exchange interactions are denoted as *J*_1_, *J*_2_, and *J*_3_, respectively. The in‐plane Néel vector is parameterized as $\hat{n}\left(\varphi \right)$, where φ is the azimuth angle. b) Side view of the G‐type AM CoPSe_3_ bilayer, with interlayer magnetic coupling labeled as *J*_c_. c) Local coordination environment of the distorted CoSe_6_ octahedron. d) Schematic illustration of spin‐exchange coupling mechanisms in the G‐type AM phase. Arrow lengths qualitatively represent the number of spin‐polarized electrons. Red and black curved arrows indicate charge transfer (solid), orbital overlap (dashed), and electron hopping (dotted). e) Layer‐resolved spin charge density difference in the ground‐state AM configuration, with an isosurface value of 8   ×  10^−5^ e·(Bohr)^−3^. Yellow and blue contours indicate regions of charge accumulation and depletion, respectively.

To explore the intrinsic mechanism of magnetic coupling for the CoPSe_3_ bilayer, we employ a spin‐Heisenberg model^[^ ^55^, ^61^ ^]^

(7) $$\begin{array}{c} {\hat{H}}_{\mathrm{spin}}=\sum_{n=1}^{3}J_{n}\sum_{\langle i,j\rangle }{\hat{S}}_{i}\cdot {\hat{S}}_{j}+J_{c}\sum_{\langle i,j\rangle }{\hat{S}}_{i}\cdot {\hat{S}}_{j} \end{array}$$

where *J_n_* (*n* = 1, 2, 3) and *J*_c_ represent the first to third nearest‐neighbor of intralayer and the nearest‐neighbor interlayer magnetic interactions, as illustrated in **Figure** **2**a,b, respectively. Here, ${\hat{S}}_{i}$ and ${\hat{S}}_{j}$ are spin operators corresponding to the magnetic moments at sites *i* and *j*. By combining the spin‐Heisenberg model with supercell mapping analysis described in Section III of Supporting Information,^[^ ^55^, ^61^ ^]^ the intralayer exchange parameters (*J*_1_ = −0.98 meV, *J*_2_ = −0.01 meV, *J*_3_ = 3.96 meV, as labeled in **Figure** 2a; Figure S2a, Supporting Information) and the interlayer exchange parameter (*J*_c_ = 0.08 meV, as labeled in **Figure** 2b; Figure S2b, Supporting Information) are extracted by systematically modulating the spin orientations at sites *i* and *j*. The sign of *J* provides insights into the nature of the interaction: positive *J* indicates AFM coupling, while negative *J* corresponds to FM coupling. Although *J*_1_ and *J*_2_ favor FM interactions, the strong AFM interactions of *J*_3_ dominate the intralayer interactions. As a result, both the intralayer magnetic coupling (driven by *J*_3_) and the interlayer coupling (determined by *J*_c_) exhibit a clear preference for AFM ordering in CoPSe_3_ bilayers.

These magnetic exchange results derived from the spin‐Heisenberg model can be understood based on the superexchange mechanism.^[^ ^62^, ^63^, ^64^ ^]^ For intralayer couplings, the bonding angle plays a critical role. The magnetic coupling between nearest‐neighbor Co atoms is weakened due to the competition between direct exchange and superexchange interactions. At the microscopic level, direct exchange arising from 3*d* orbital overlap typically favors AFM coupling.^[^ ^65^ ^]^ However, as shown in Figure S3a (Supporting Information), the Co─Se─Co bond angle of ≈87° favors FM superexchange according to the Goodenough–Kanamori–Anderson (GKA) rules.^[^ ^62^, ^63^, ^64^ ^]^ These competing mechanisms lead to a weak FM interaction between nearest‐neighbor Co atoms, with an exchange constant of *J*_1_ = −0.98 meV. In contrast, the magnetic coupling between third‐neighbor Co atoms is predominantly governed by superexchange interactions. As shown in Figure S3b (Supporting Information), the Co─P─Se bond angle of ≈102° in the P_2_Se_6_ coordination environment deviates from the critical 90°, thereby favoring strong AFM coupling as governed by GKA rules.^[^ ^66^ ^]^ As a result, this interaction with *J*_3_ = 3.96 meV determines the overall intralayer AFM configuration.

For interlayer exchange coupling, it is mainly attributed to interfacial orbital overlap, which facilitates superexchange pathways (see Section SIV, Supporting Information for a detailed discussion). Although such couplings are typically weak, recent studies have revealed their significant influence on electronic properties.^[^ ^67^, ^68^, ^69^ ^]^ As illustrated in **Figure** 2c,d, the Co‐3*d* orbitals undergo *e*_g_/*t*_2g_ splitting due to the octahedral crystal field. In the G‐type AM configuration, notable orbital overlap occurs between the Se‐4*p* orbitals at the interlayer interface. This overlap involves opposite spin components, as evidenced by the layer‐resolved spin differential charge density in **Figure** 2e. The resulting spin configuration enables antiparallel spin charge transfer between layers, which mediates AFM coupling. By comparison, for the C‐type AFM configuration (Figure S8, Supporting Information), the orbital overlap occurs between the same spin states, leading to FM coupling. When the overlapping electrons share the same spin, the enhanced Pauli repulsion increases the system's energy. In contrast, the overlap between opposite spin electrons reduces this repulsion, favoring AFM coupling and stabilizing the G‐type AM phase as the magnetic ground state of the CoPSe_3_ bilayer. Nevertheless, a phase transition between the G‐type AM and C‐type AFM configurations can be realized by applying an electric field (see Figure S9, Supporting Information for details), which provides a feasible and tunable route to modulate interlayer magnetic coupling in CoPSe_3_ bilayer for potential spintronic applications.

**Figure 3:**
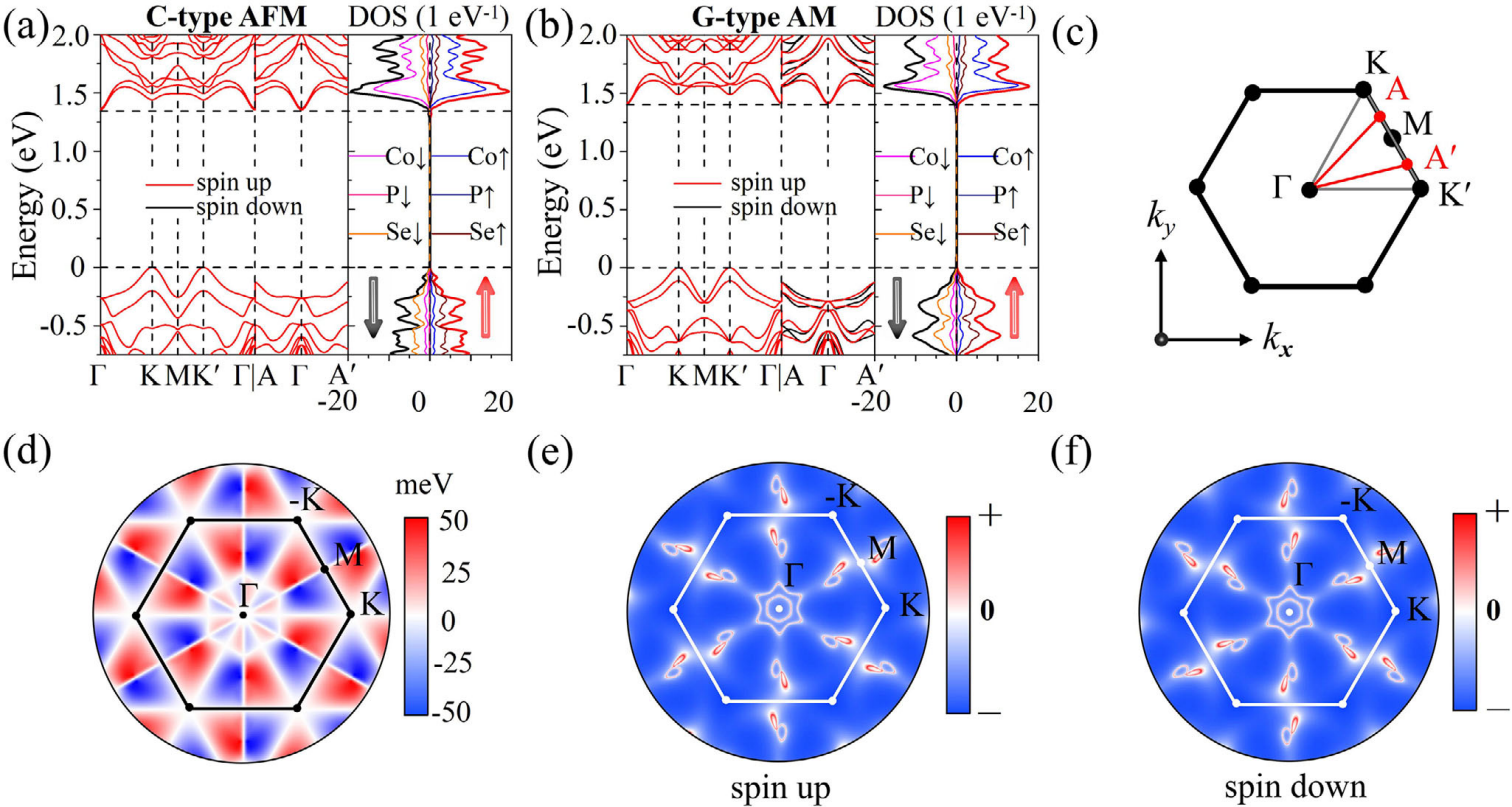
Band structures of CoPSe_3_ bilayer in the a) C‐type AFM and b) G‐type AM configurations along high‐symmetric *k*‐path (Γ‐K‐M‐K*'*‐Γ) and generic *k*‐path (A‐Γ‐A*'*). The corresponding projected DOS is shown alongside each plot. c) First Brillouin zone of the hexagonal lattice with high‐symmetry points labeled as Γ, M, K/K*'*, and generic points A/A*'*; A/A*'* mark the midpoints between M and K/K*'*. d) Spin splitting distribution (*E*_↑_  −  *E*_↓_) for the valence band without SOC. Fermi surface for e) spin‐up and f) spin‐down states, with the Fermi level set to −0.29 eV below the VBM.

In **Figure** **3**a,b, we display the electronic structures of C‐type and G‐type CoPSe_3_ bilayers without incorporating relativistic spin–orbit coupling (SOC). Both configurations exhibit Kramers spin degeneracy along the high‐symmetric *k*‐path (Γ‐K‐M‐K*'*‐Γ in **Figure** 3c). However, distinct differences emerge along the generic *k*‐path (A‐Γ‐A*'*). In the C‐type AFM configuration, the energy bands maintain spin degeneracy along both the high‐symmetric and generic *k*‐paths. In contrast, the G‐type AM configuration exhibits pronounced spin splitting for a generic *k*, even in the absence of relativistic SOC. Furthermore, while spin alternates in momentum space, the net magnetization remains zero, as clearly demonstrated by its density of states (DOS). To clarify the underlying mechanism, we carry out a symmetry analysis of the CoPSe_3_ bilayer. The AA‐stacked CoPSe_3_ bilayer in its nonmagnetic state belongs to the space group $P\bar{3}1m$ (no. 162) and point group *D*_3*d*_, which comprises a total of 12 symmetry operations. These operations form the set $\left\{E,\;P,2C_{3},2S_{6},3M_{d},3C_{2}\right\}$, including identity representation E, inversion symmetry P, two 3‐fold rotation symmetries $C_{3}$, two 6‐fold rotation‐reflection symmetries $S_{6}$, three mirror symmetries $M_{d}$, and three 2‐fold rotation symmetries $C_{2}$.

Upon introducing collinear AFM order, both crystal and spin rotation symmetries should be considered, which remain decoupled in the absence of SOC. Notably, the spin can be treated within the framework of the spin group theory,^[^ ^70^, ^71^ ^]^ which provides a comprehensive description of the symmetries for magnetic materials without SOC (see Section VII in Supporting Information). For simplicity, we treat the spin as a pseudoscalar,^[^ ^72^ ^]^ in which spin remains invariant under spatial symmetry operations but reverses sign under the antisymmetric operator $R_{S}$. This operator plays the role of a non‐relativistic analog of time‐reversal symmetry, relevant for spin‐resolved systems without SOC. Therefore, when considering the AM order, the symmetry operations can be expressed as:

(8) $$\begin{array}{c} \left\{E,\;P,2C_{3},2S_{6}\right\}+R_{S}\left\{3M_{d},3C_{2}\right\} \end{array}$$

Here, the operations in the first set preserve each magnetic sublattice, while those in the second set exchange the two spin‐opposed sublattices. For example, under the symmetry operation $R_{S}M_{d}^{x}$, the system satisfies the relation:

(9) $$\begin{array}{c} R_{S}M_{d}^{x}E\left(k_{x},k_{y},↑\right)=E\left(-k_{x},k_{y},↓\right) \end{array}$$

where ↑ and ↓ denote different spin components. At *k_x_* = 0, Equation (9) reduces to *E*(0,  *k_y_*, ↑) = *E*(0,  *k_y_*, ↓), which implies that the band energies for opposite spins are degenerate along the *k_x_* = 0 path (represented by the red solid line in Figure S10a, Supporting Information). The other two mirrors $M_{d}$ generate equivalent spin‐degenerate paths, spaced at 60° intervals as indicated by the red dashed lines in Figure S10a (Supporting Information). Similarly, applying the symmetry $R_{S}C_{2}^{x}$ leads to the relation:

(10) $$\begin{array}{c} R_{S}C_{2}^{x}E\left(k_{x},k_{y},↑\right)=E\left(k_{x},-k_{y},↓\right) \end{array}$$

when *k_y_* = 0, this simplifies to *E*(*k_x_*, 0,  ↑) = *E*(*k_x_*, 0,  ↓), indicating a spin‐degenerate line along *k_y_* = 0, as shown by the blue solid line in Figure S10b (Supporting Information). The remaining two $C_{2}$ operations generate additional spin‐degenerate lines, again separated by 60° intervals as indicated by the blue dashed lines in Figure S10b (Supporting Information).

By combining all these symmetry‐protected paths with Bloch's theorem, we identify all symmetry‐enforced spin‐degenerate *k*‐paths within the first Brillouin zone. These paths, along which spin splitting is strictly forbidden by symmetries, are depicted as white lines in **Figure** 3d. In contrast, away from these high‐symmetry directions, local variations in the magnetization density around the Co sublattices break the symmetry protection and lift the degeneracy, leading to nonrelativistic spin splitting. This effect is visualized in **Figure** 3d, which maps the spin splitting as *E*_↑_  −  *E*_↓_ in the valence band across the Brillouin zone. The results reveal that the spin splitting reaches its maximum (up to 50 meV) on both sides of the Γ‐M path. In addition, the spin‐split Fermi surfaces shown in **Figure** 3e,f provide further evidence of spin polarization and symmetry breaking. These results demonstrate that the incorporation of spin degrees of freedom, together with interlayer magnetic coupling in vdW materials, can break symmetry and induce new physical phenomena within the homogeneous hexagonal system.

**Figure 4:**
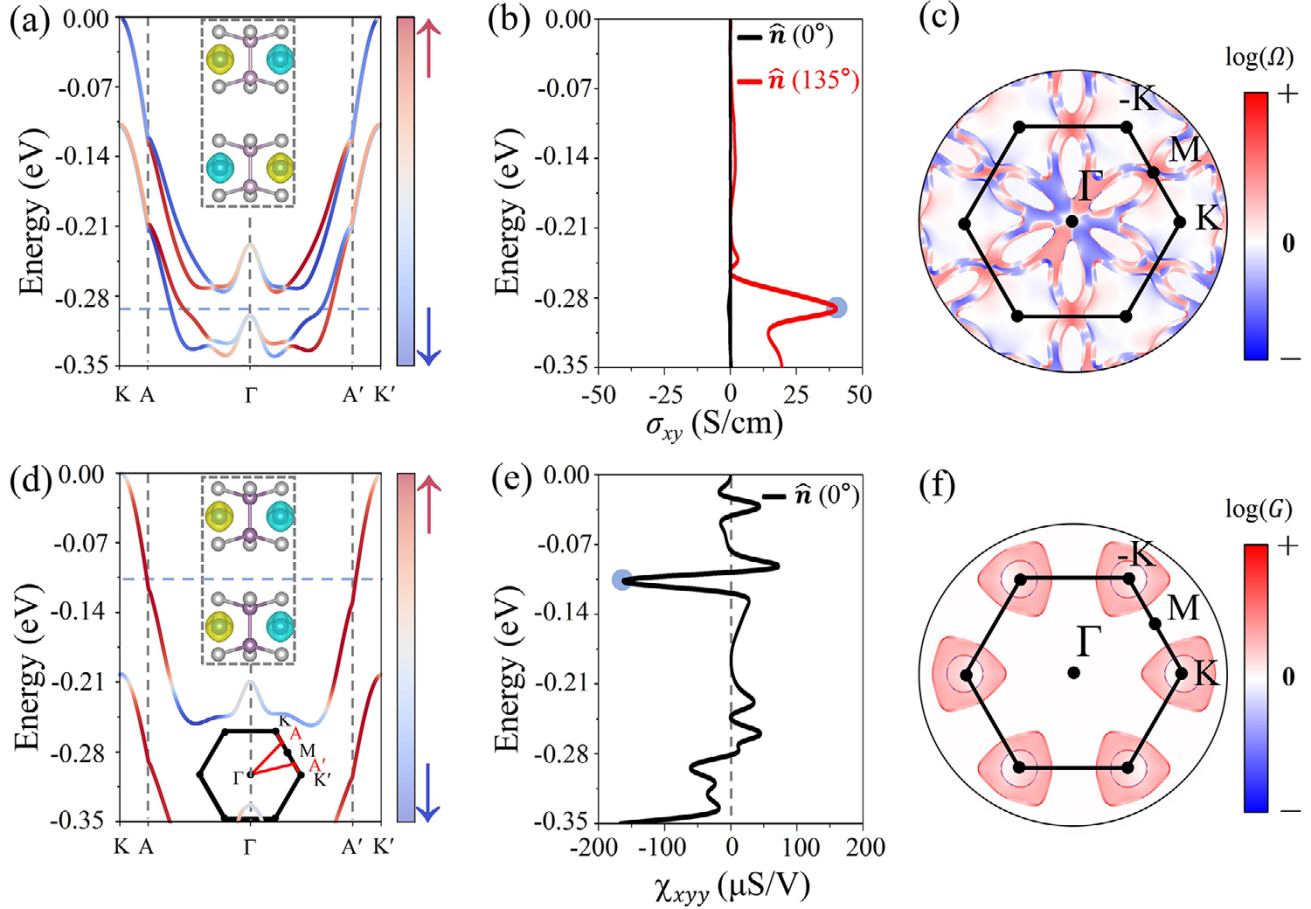
a) Spin‐resolved band structure of the G‐type AM CoPSe_3_ bilayer with SOC. b) Intrinsic AHC as a function of the Fermi energy for the G‐type AM phase. The first peak, located at −0.29 eV below the VBM, is marked by a blue bubble. c) Corresponding *k*‐resolved Berry curvature distribution in the 2D Brillouin zone. d) Spin‐resolved band structure of the C‐type AFM CoPSe_3_ bilayer with SOC. e) The second‐order NAHE as a function of the Fermi energy for the C‐type AFM phase. The peak highlighted by the blue bubble is located at −0.11 eV below the VBM. f) Corresponding *k*‐resolved BCP in the 2D Brillouin zone. A logarithmic transformation, as described in the Computational Details, is applied to both the Berry curvature and BCP. The VBM is set to 0 eV.

The emerging spin structures in G‐type AM CoPSe_3_ bilayer offer opportunities for magnetoelectronic responses, providing a direct method for electric control of magnetism. To elucidate this phenomenon, we explore the AHE in the AM CoPSe_3_ bilayer by including the SOC effect. As illustrated in **Figure** **4**b, the system exhibits pronounced anisotropy in the AHC with respect to the orientation of the Néel vector. When the Néel vector is aligned along $\hat{n}\left(0^{\circ }\right)$, the system retains its magnetic point group (MPG) 2/m.1, which imposes 2D nature and symmetry restrictions that prohibit the occurrence of the AHE. However, when the Néel vector is oriented along $\hat{n}\left(135^{\circ }\right)$, the symmetry is lowered to MPG 2'/m', allowing a nonzero AHC component σ_*xy*_. In this case, the AHC peaks at 46 S cm^−1^ at an energy of −0.29 eV below the valence band maximum (VBM), as highlighted by the blue bubble in **Figure** 4b. The spin‐resolved band structure of AM CoPSe_3_ shown in **Figure** 4a, provides further insight into the origin of this AHC behavior. When the SOC is taken into account, the bilayer exhibits spin splitting with remarkable (anti)crossings at the Γ‐point. Note that this crossing point highlighted by a color projection is not a coincidence but is determined by the alternating characteristic of spin polarization in momentum space.^[^ ^22^, ^73^ ^]^ Evidently, these crossings contribute significantly to the AHC peak observed in **Figure** 4b.

In **Figure** 4c, we show the *k*‐dependent Berry curvature distribution associated with the peak in **Figure** 4b. The results reveal that the localized Berry curvature along the generic *k*‐path directly contributes to the nonzero AHC, enabling the generation of a transverse current under an external electric field. The AHE is absent when the Néel vector is oriented along the *z*‐axis (out of the plane), regardless of the magnetic phase. However, it emerges distinctly when the Néel vector lies within the intralayer plane of the AM phase, emphasizing the dependence of the AHE on both the magnetic phase and the Néel vector orientation. Relevant details regarding the symmetry restrictions and anisotropic AHE across different magnetic configurations are summarized in Tables S2–S4 (Supporting Information).

As discussed above, the CoPSe_3_ bilayer with the C‐type AFM phase exhibits PT‐symmetry, which enforces spin degeneracy and suppresses the linear AHE (see **Figures** 3a and 4d). Nevertheless, a higher‐order anomalous Hall signal can still emerge in this phase. Notably, due to the preserved PT‐symmetry, the Berry curvature dipole (BCD) contribution is symmetry‐forbidden in this phase, rendering the quantum metric the dominant source of the second‐order response.^[^ ^45^, ^46^ ^]^ A detailed symmetry analysis is provided in Section VIII of the Supporting Information. In **Figure** 4c, we present the NAHC induced by the quantum metric as a function of Fermi energy. The peak (χ_*xyy*_ ≈ 160 µS V^−1^) highlighted by the blue bubble in the AFM CoPSe_3_ bilayer is comparable to that of bulk antiferromagnet such as Mn_2_Au (∼150 µS V^−1^) in reference.^[^ ^45^ ^]^ This demonstrates the remarkable potential of the CoPSe_3_ bilayer for nonlinear magnetoelectric applications.

In **Figure** 4f, we present the *k*‐dependent distribution of BCP, corresponding to the peak marked by the blue bubble in **Figure** 4e. In contrast to the Berry curvature distribution associated with the linear AHC in the AM phase, the BCP in the C‐type AFM phase is concentrated primarily near high‐symmetric *k*‐points. Specifically, the second‐order NAHE is predominantly governed by the localized BCP at the K‐point, highlighting its critical role in driving the observed nonlinear Hall response. Importantly, since the second‐order NAHE holds the potential to significantly enhance detector responses and improve terahertz spectroscopic imaging, it presents a compelling opportunity for advancements in terahertz and infrared technologies.^[^ ^74^, ^75^ ^]^ Thus, the hexagonal CoPSe_3_ bilayer, with tunable large anisotropic AHE and second‐order NAHE via interlayer magnetic coupling, provides a promising platform for integrated devices.

According to the symmetry analysis, nonrelativistic spin‐momentum coupling can occur not only in bilayer systems but also in the few‐layer systems. As demonstrated by the AA‐stacked trilayer structure shown in Figure S11 (Supporting Information), where the PT‐ symmetry is preserved in both C‐type AFM and G‐type AFM configurations but broken in the hybrid magnetic configuration. Figure S12 (Supporting Information) further verifies the nonrelativistic spin splitting in the trilayer system. Therefore, our findings in the CoPSe_3_ bilayer can be naturally extended to few‐layer systems.

## 4 Conclusion

In summary, we unveil a quantum geometric paradigm for realizing dual Hall effects in 2D CoPSe_3_ bilayers via interlayer magnetic coupling. The AM phase generates a robust anisotropic AHE governed by Berry curvature hotspots, while the PT‐symmetric AFM phase exhibits an intrinsic second‐order NAHE dominated by quantum metric contributions at high‐symmetric *k*‐points. The reversible transition between these phases, mediated by interlayer exchange interactions, underscores the critical role of quantum geometry in linking band structures to transport phenomena. Beyond demonstrating the coexistence of linear and nonlinear Hall responses, our findings reveal how symmetry engineering in layered antiferromagnets can selectively activate distinct quantum geometric mechanisms. The integration of AHE and NAHE in a single material system opens avenues for multifunctional device architectures, where quantum geometry serves as a cornerstone for emergent magnetoelectric functionalities.

## Conflict of Interest

The authors declare no conflict of interest.

## Supporting information



Supporting Information

## Data Availability

The data that support the findings of this study are available from the corresponding author upon reasonable request.
