# Supplementary material for: Quantum Geometric Engineering of Dual Hall Effects in 2D Antiferromagnetic Bilayers via Interlayer Magnetic Coupling
Source: Adv Sci (Weinh). 2025 May 28;12(31):e05860. doi: 10.1002/advs.202505860 (PMC12376605; doi:10.1002/advs.202505860)
Supplement: Supplementary file 1 — Supporting Information [file ADVS-12-e05860-s001.pdf]

ADVANCED SCIENCE
Open Access

Supporting Information

for *Adv. Sci.*, DOI 10.1002/advs.202505860

Quantum Geometric Engineering of Dual Hall Effects in 2D Antiferromagnetic Bilayers via Interlayer Magnetic Coupling

Zhenning Sun, Tao Wang, Hao Jin, Xinru Li, Yadong Wei* and Jian Wang*

Supporting Information

Quantum Geometric Engineering of Dual Hall Effects in 2D Antiferromagnetic Bilayers via Interlayer Magnetic Coupling

Zhenning Sun[1], Tao Wang[1], Hao Jin[1], Xinru Li[2], Yadong Wei[1]*, Jian Wang[1,3]*

[1]*College of Physics and Optoelectronic Engineering, Shenzhen University, Shenzhen 518060, P. R. China*

[2]*School of Physics, State Key Laboratory of Crystal Materials, Shandong University, Shandanan Street 27, Jinan 250100, China*

[3]*Department of Physics, The University of Hong Kong, Pokfulam Road, Hong Kong 999077, China*

CONTENTS

I. Geometric Structures S-3

II. Convergence Tests and Thermodynamic Stability S-4

III. Supercell Mapping Analysis S-6

IV. Interlayer Magnetic Coupling S-7

V. Spin-exchange Coupling Mechanisms for C-type AFM Phase S-8

VI. Phase Transition via Electric Field S-10

VII. Spin Space Group Analysis S-11

VIII. Symmetry Analysis for Non-relativistic Spin Splitting S-12

IX. $\mathcal{PT}$-symmetry Exclusion of BCD in NAHE S-13

X. Anisotropy of the AHE and the NAHE S-14

XI. Spin Splitting in the Trilayer System S-16

XII. References S-18

I. Geometric Structures

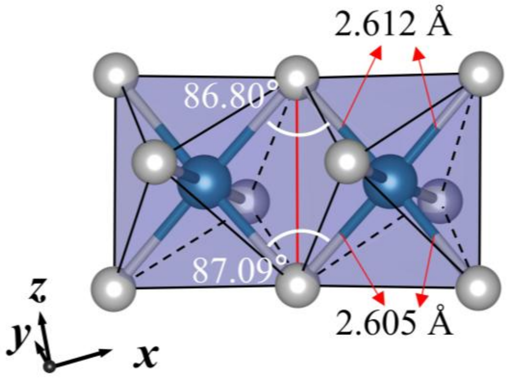

Figure S1. Local structure of neighboring $CoSe_6$ octahedra. The solid red line is the boundary of the neighboring octahedra.

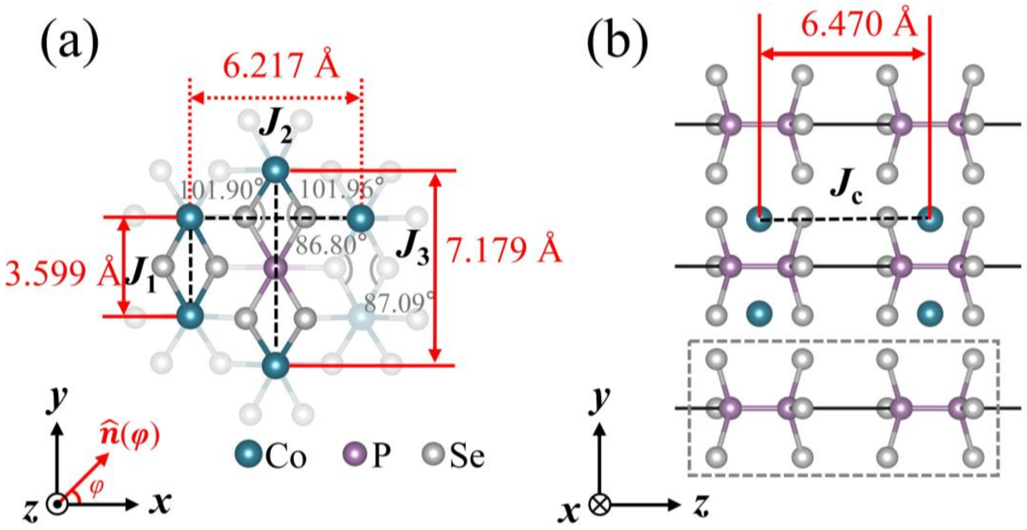

Figure S2. a) Geometric information among magnetic atoms with a) intralayer magnetic coupling coefficients J_1, J_2, and J_3, and b) interlayer magnetic coupling coefficient J_c.

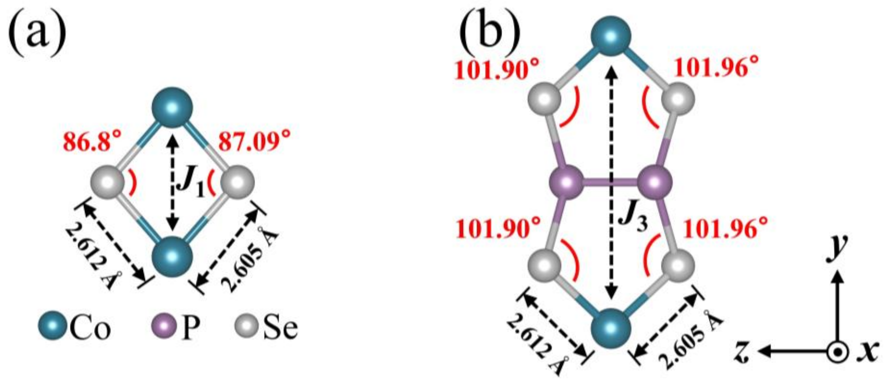

Figure S3. Bond length and bond angle for $CoPSe_3$ bilayer.

II. Convergence Tests and Thermodynamic Stability

Convergence tests are systematically performed to validate parameter selection and ensure computational stability. The cutoff energy analysis reveals stable convergence for energies above 500 eV. Additionally, *k*-point grid testing shows that grids finer than 8 × 8 × 1 produce consistent results. Consequently, optimal parameters are established as a 500 eV cutoff energy and a 12 × 12 × 1 *k*-point grid, as labeled by the dashed lines in **Figure S4**.

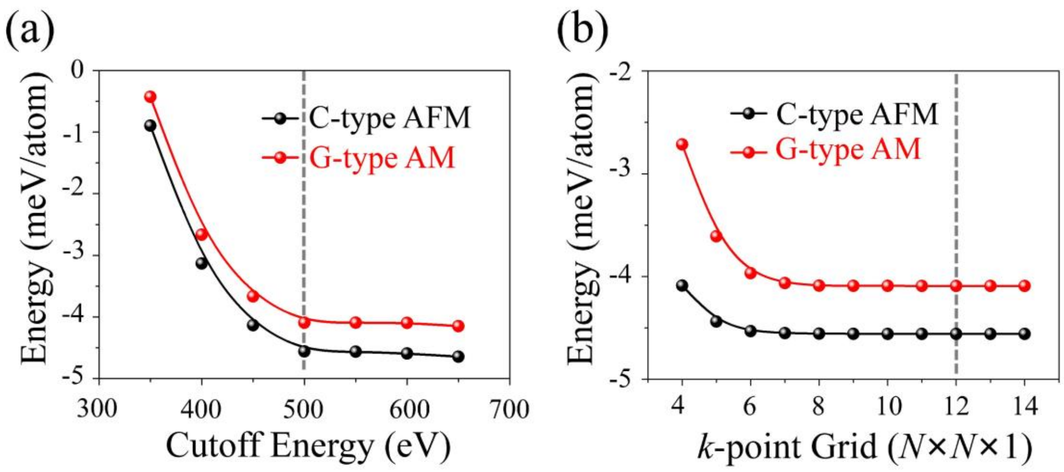

Figure S4. Relative energy differences as a function of a) cutoff energy and b) *k*-point grids.

To validate the robustness of our result, we have performed a series of convergence tests by varying key computational parameters, including the supercell size, plane-wave cutoff energy, and *k*-point sampling. As shown in **Figure S5**, across all tested conditions, the resulting values of J_c vary by less than 0.01 meV. This confirms that the calculated value of $J_c \approx 0.08$ meV is numerically stable and well-converged with respect to the key DFT parameters. Therefore, we consider the reported J_c value to be quantitatively reliable within a numerical uncertainty of ±0.01 meV.

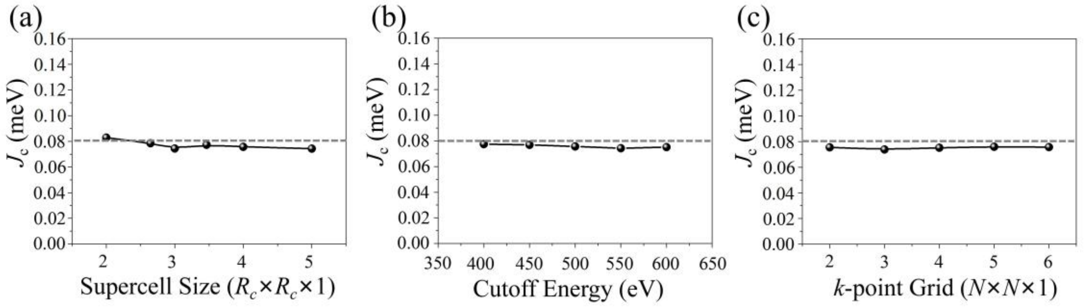

Figure S5. Interlayer magnetic coupling J_c as a function of a) supercell size, b) cutoff energy, and c) k-point grids.

Ab initio molecular dynamics (AIMD) simulations using a 3 × 3 × 1 supercell at 300 K are performed to investigate the thermodynamic stability of the $CoPSe_3$ bilayer. The results shown in **Figure S6** confirm that the $CoPSe_3$ bilayer retains its structural integrity throughout the simulation, with no bond breaking or significant lattice distortions observed. Moreover, the G-type AM configuration remains the ground state, with no evidence of a phase transition during the simulation. These findings confirm that the energy differences between the G-type AM and C-type AFM phases are sufficient to ensure the practical phase stability required for reversible phase switching in device applications.

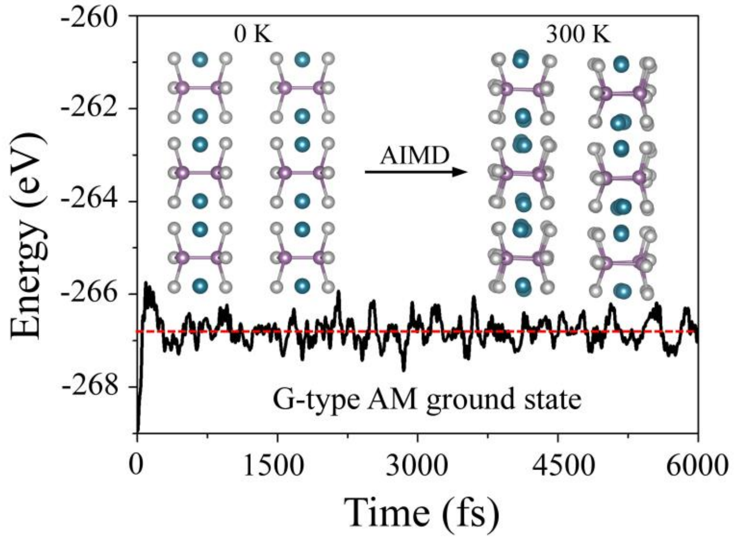

Figure S6. Time evolution of free energy during the AIMD simulation at 300 K.

III. Supercell Mapping Analysis

A detailed description of the methodology for extracting the magnetic exchange parameters J_1, J_2, J_3, and J_c from first-principles calculations is provided below. The extraction of exchange parameters is based on a four-state spin-mapping approach.[1-2] In this work, we consider a 4 × 4 × 1 supercell and select two magnetic ions at sites A and B whose relative spin orientation is varied, while keeping all other spins fixed in their ground-state orientations. By choosing different A-B pairs corresponding to first-, second-, third-, and interlayer neighbors, we can isolate and evaluate the corresponding exchange constants J_1, J_2, J_3, and J_c, respectively. The total energy is expressed as:

$$E = E_0 + E_{\text{spin}} = E_0 + J_{\text{AB}}\boldsymbol{S}_{\mathbf{A}} \cdot \boldsymbol{S}_{\mathbf{B}} + \boldsymbol{S}_{\mathbf{A}} \cdot \boldsymbol{K}_{\mathbf{A}} + \boldsymbol{S}_{\mathbf{B}} \cdot \boldsymbol{K}_{\mathbf{B}} + E_{\text{other}}, \qquad (S-1)$$

where E_0 represents nonmagnetic reference energy. $J_{\text{AB}}\boldsymbol{S}_{\mathbf{A}} \cdot \boldsymbol{S}_{\mathbf{B}}$ is the exchange interaction between spins at A and B sites. $\boldsymbol{S}_{\mathbf{A/B}} \cdot \boldsymbol{K}_{\mathbf{A/B}}$ accounts for interactions between A/B ions and all other magnetic ions except B/A ions. The terms $\boldsymbol{K}_{\mathbf{A}}$ and $\boldsymbol{K}_{\mathbf{B}}$ are defined as $\boldsymbol{K}_{\mathbf{A}} = \sum_{N \neq \text{A,B}} J_{\text{A}N}\boldsymbol{S}_{\boldsymbol{N}}$ and $\boldsymbol{K}_{\mathbf{B}} = \sum_{N \neq \text{A,B}} J_{\text{B}N}\boldsymbol{S}_{\boldsymbol{N}}$, respectively. E_{other} includes constant terms from the unaltered spins. To determine the exchange interactions J_{AB}, we construct four distinct collinear spin configurations: ($\boldsymbol{S}_{\mathbf{A}}$, $\boldsymbol{S}_{\mathbf{B}}$, $\boldsymbol{S}_{\mathbf{other}}$), ($\boldsymbol{S}_{\mathbf{A}}$, -$\boldsymbol{S}_{\mathbf{B}}$, $\boldsymbol{S}_{\mathbf{other}}$), (-$\boldsymbol{S}_{\mathbf{A}}$, $\boldsymbol{S}_{\mathbf{B}}$, $\boldsymbol{S}_{\mathbf{other}}$), and (-$\boldsymbol{S}_{\mathbf{A}}$, -$\boldsymbol{S}_{\mathbf{B}}$, $\boldsymbol{S}_{\mathbf{other}}$).

By subtracting energies of the four configurations, terms involving $K_{\text{A/B}}$ and E_{other} cancel out, yielding:

$$J_{\text{AB}} = \frac{E_1 + E_4 - E_2 - E_3}{4\boldsymbol{S}_{\mathbf{A}} \cdot \boldsymbol{S}_{\mathbf{B}}}, \qquad (S-2)$$

where E_{1-4} are total energies of the constructed configurations, which can be directly obtained from our DFT calculations. Consequently, the magnetic coupling interactions (J_{1-3} and J_c) between different neighbors can be obtained by mapping lattice sites A and B to distinct adjacent atoms.

IV. Interlayer Magnetic Coupling

We have analyzed the dependence of J_c on the interlayer distance d, as well as the differential charge density distribution between the two layers. As shown in **Figure S7a**, J_c exhibits an exponential decay with increasing interlayer distance ($J_c \propto 59.11e^{-2.06d}$), characteristic of orbital-mediated exchange interactions. Correspondingly, the differential charge density in **Figure S7b** shows reduced interlayer charge accumulation at larger d, suggesting weakened electronic hybridization across the interface.

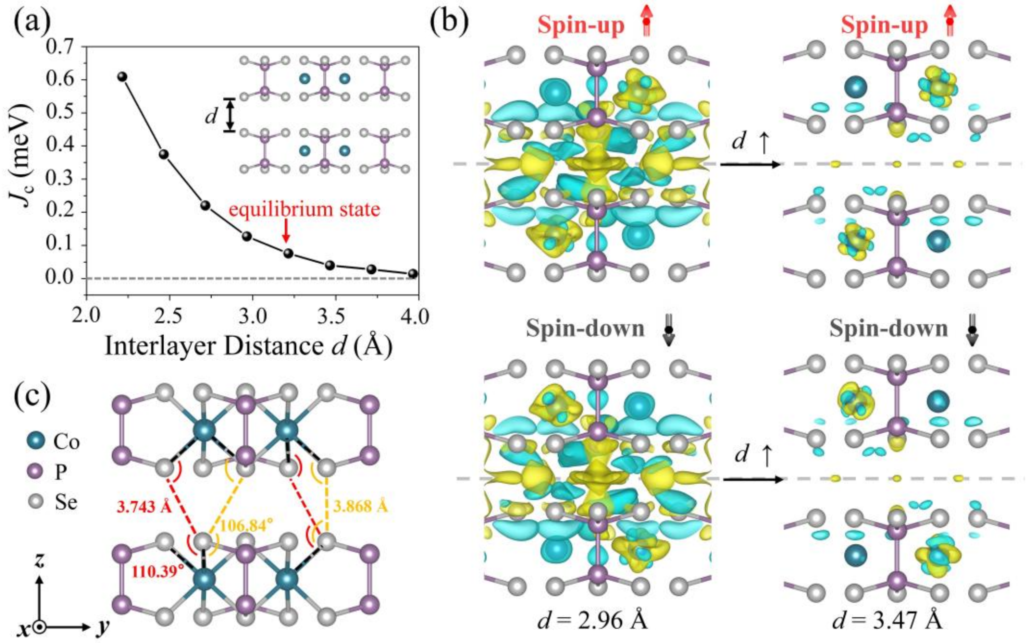

Figure S7. a) Interlayer magnetic coupling J_c as a function of interlayer distance. b) The bond angle between interlayer Co atoms and the interlayer distance between Se atoms marked with different colors. c) Side view of the layer-resolved difference in charge density for spin-up and spin-down electrons in the G-type AM $CoPSe_3$ bilayer, with interlayer distance d as 2.96 Å and 3.47 Å, respectively. The isosurface value is selected to be 7.5×10^{-5} e·(Bohr)$^{-3}$. Yellow and blue contours indicate regions of charge accumulation and depletion, respectively.

These results demonstrate that although van der Waals (vdW) interactions determine the equilibrium interlayer spacing, the interlayer magnetic coupling is primarily driven by interfacial orbital overlap, which enables superexchange pathways. In this system, the magnetic coupling between Co atoms in adjacent layers is mainly mediated via the Co-Se-Se-Co pathway, involving virtual hopping of electrons. Similar to the intralayer interactions, the bonding angle along this path plays a crucial

role. As shown in **Figure S7c**, the Co-Se-Se bond angle is approximately 110° with a shorter length of Se-Se, which favors AFM coupling according to the GKA rules. Notably, similar interlayer superexchange mechanisms have been reported in other vdW bilayers such as CrI_3[3] and $CrSe_2$[4], reinforcing the generality of this behavior.

V. Spin-exchange Coupling Mechanisms for C-type AFM Phase

For comparison, **Figure R8** illustrates the spin-exchange coupling mechanism in the C-type AFM configuration. At the interlayer interface, the Se-4*p* orbitals also exhibit overlap, as evidenced by the layer-resolved spin charge density difference shown in **Figure R8e**. However, unlike the G-type AM phase, the overlap in the C-type configuration involves orbitals of the same spin component. This is indicated by the black arrows in the black-colored region of **Figure R8d**, where the arrow lengths qualitatively represent the number of spin-polarized electrons. Consequently, electrons with the same spin from Co-3*d* orbitals in different layers are transferred into the overlapping region, which mediates FM coupling. Notably, when the overlapping electrons share the same spin, enhanced Pauli repulsion increases the system's energy. In contrast, opposite-spin overlap minimizes repulsion, favoring AFM coupling and stabilizing the G-type AM phase as the magnetic ground state of the $CoPSe_3$ bilayer.

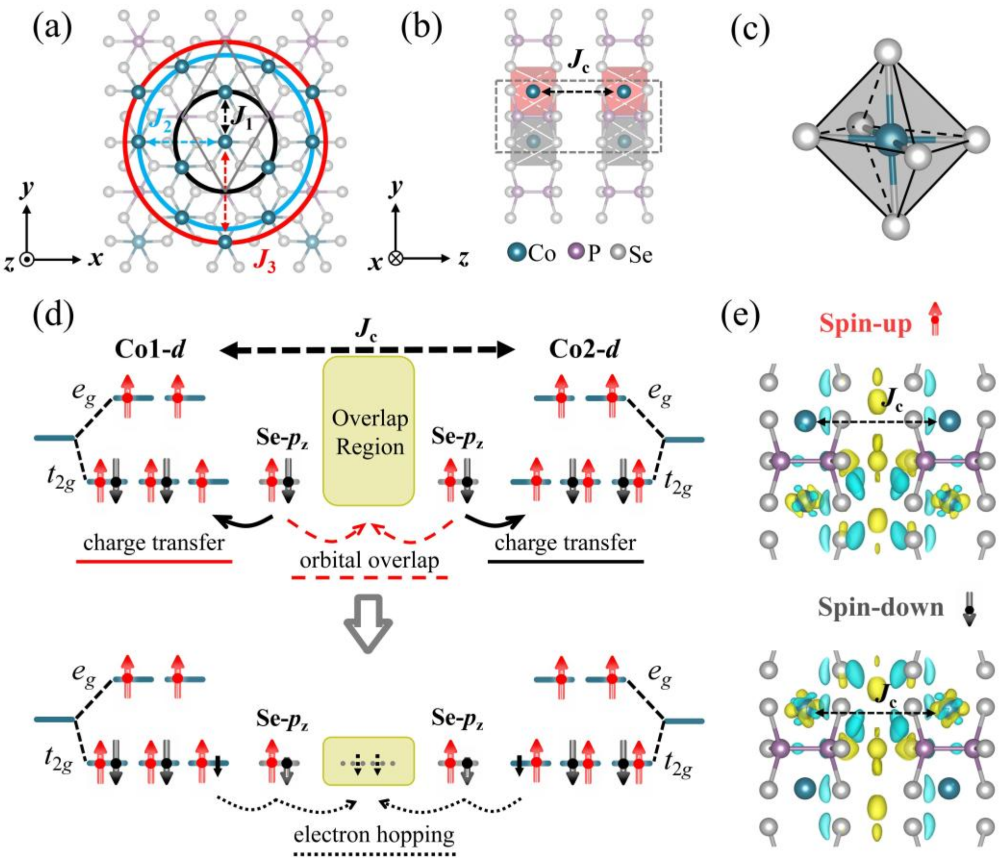

Figure S8. a) Top view of $CoPSe_3$ bilayer, with the primitive cell outlined by a solid gray diamond. The nearest second-nearest, and third-nearest neighbors of the central Co atom are marked with black, blue, and red circles, respectively. The corresponding intralayer magnetic couplings are labeled as J_1, J_2, and J_3, respectively. The in-plane Néel vector is parameterized as $\hat{n}(\varphi)$, where φ is the azimuth angle. b) Side view of C-type AFM $CoPSe_3$ bilayer, with the interlayer magnetic coupling labeled as J_c. c) Local coordination environment of the distorted $CoSe_6$ octahedron. d) Schematic illustration of spin-exchange coupling mechanisms in the C-type AFM phase. Arrow lengths qualitatively represent the number of spin-polarized electrons. Red and black curved arrows indicate charge transfer (solid lines), orbital overlap (dashed lines), and electron hopping (dotted lines). e) Layer-resolved spin charge density difference in the ground-state AM configuration, with an isosurface value of 8×10^{-5} e·(Bohr)$^{-3}$. Yellow and blue contours indicate regions of charge accumulation and depletion, respectively.

VI. Phase Transition via Electric Field

Applying the electric field is a common tuning method in experiments.[5-6] At present, dual ionic gating has been experimentally demonstrated to achieve an intense electric field larger than 0.4 V Å^{-1}.[7] Given the significant influence of charge transfer on interlayer magnetic coupling interactions within the spin-exchange coupling mechanism in our results, we have investigated the magnetic phase manipulation of $CoPSe_3$ bilayers using an electric field perpendicular to the material plane. F**igure S9** illustrates the relationship between the energy difference $E_{\mathrm{AM-AFM}}$ and the electric field E. It shows that the bilayer system undergoes a transition from the G-type AM phase to the C-type AFM phase at a critical electric field of 0.355 V Å^{-1}. Therefore, within the experimentally accessible range, it is feasible to achieve the transition between two zero-net-magnetization phases in the bilayers via an electric field, thereby facilitating the control of dual Hall responses in device applications.

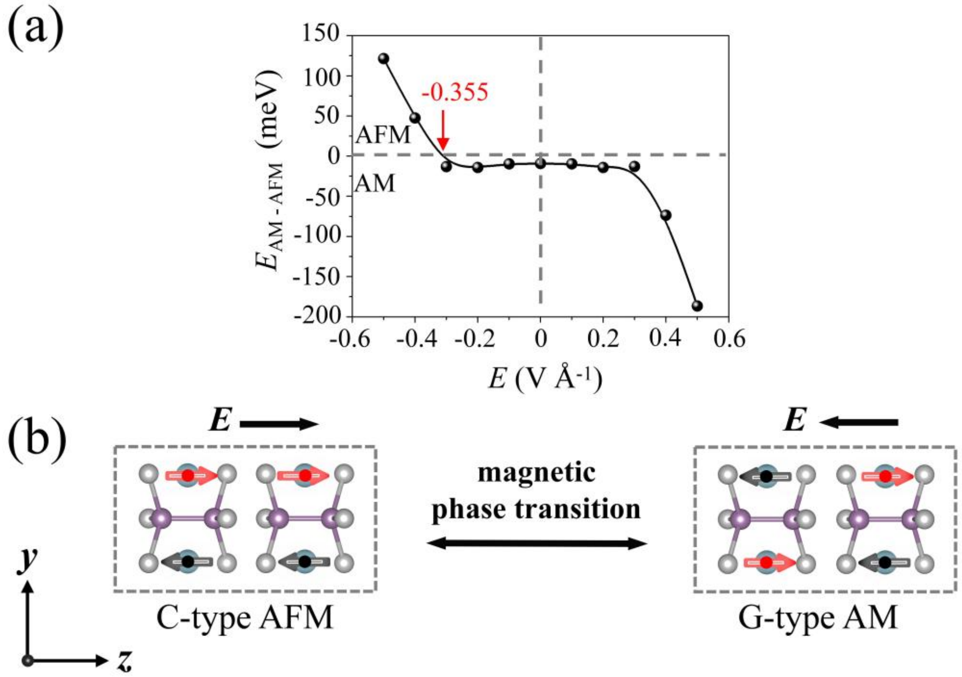

Figure S9. a) Energy difference $E_{\mathrm{AM-AFM}}$ as a function of the electric field E. b) Schematic diagram of reversible magnetic phase transition under an electric field E.

VII. Spin Space Group Analysis

The treatment of spin in the context of spin space group (SSP) can provide a more comprehensive and accurate description of the symmetry properties for magnetic materials without SOC. The symmetry operations described in **Eq. 8** of the manuscript can then be rewritten as follows:

Table S1. Elements of the spin space group for G-type AM phase.

No.	Spin Rotation	Space Rotation	Space Translation	Seitz Symbol
1	$\begin{bmatrix}1.0 & 0.0 & 0.0\\0.0 & 1.0 & 0.0\\0.0 & 0.0 & 1.0\end{bmatrix}$	$\begin{bmatrix}1.0 & 0.0 & 0.0\\0.0 & 1.0 & 0.0\\0.0 & 0.0 & 1.0\end{bmatrix}$	$\begin{bmatrix}0.0\\0.0\\0.0\end{bmatrix}$	$\{1\|\|1\|\tau_{(0.0,0.0,0.0)}\}$
2	$\begin{bmatrix}1.0 & 0.0 & 0.0\\0.0 & 1.0 & 0.0\\0.0 & 0.0 & 1.0\end{bmatrix}$	$\begin{bmatrix}-1.0 & 0.0 & 0.0\\0.0 & -1.0 & 0.0\\0.0 & 0.0 & -1.0\end{bmatrix}$	$\begin{bmatrix}0.0\\0.0\\0.0\end{bmatrix}$	$\{1\|\|\bar{1}\|\tau_{(0.0,0.0,0.0)}\}$
3	$\begin{bmatrix}1.0 & 0.0 & 0.0\\0.0 & 1.0 & 0.0\\0.0 & 0.0 & 1.0\end{bmatrix}$	$\begin{bmatrix}0.0 & -1.0 & 0.0\\1.0 & -1.0 & 0.0\\0.0 & 0.0 & 1.0\end{bmatrix}$	$\begin{bmatrix}0.0\\0.0\\0.0\end{bmatrix}$	$\{1\|\|3^{1}_{001}\|\tau_{(0.0,0.0,0.0)}\}$
4	$\begin{bmatrix}1.0 & 0.0 & 0.0\\0.0 & 1.0 & 0.0\\0.0 & 0.0 & 1.0\end{bmatrix}$	$\begin{bmatrix}-1.0 & 1.0 & 0.0\\-1.0 & 0.0 & 0.0\\0.0 & 0.0 & 1.0\end{bmatrix}$	$\begin{bmatrix}0.0\\0.0\\0.0\end{bmatrix}$	$\{1\|\|3^{2}_{001}\|\tau_{(0.0,0.0,0.0)}\}$
5	$\begin{bmatrix}1.0 & 0.0 & 0.0\\0.0 & 1.0 & 0.0\\0.0 & 0.0 & 1.0\end{bmatrix}$	$\begin{bmatrix}0.0 & 1.0 & 0.0\\-1.0 & 1.0 & 0.0\\0.0 & 0.0 & -1.0\end{bmatrix}$	$\begin{bmatrix}0.0\\0.0\\0.0\end{bmatrix}$	$\{1\|\|\bar{3}^{1}_{001}\|\tau_{(0.0,0.0,0.0)}\}$
6	$\begin{bmatrix}1.0 & 0.0 & 0.0\\0.0 & 1.0 & 0.0\\0.0 & 0.0 & 1.0\end{bmatrix}$	$\begin{bmatrix}1.0 & -1.0 & 0.0\\1.0 & 0.0 & 0.0\\0.0 & 0.0 & -1.0\end{bmatrix}$	$\begin{bmatrix}0.0\\0.0\\0.0\end{bmatrix}$	$\{1\|\|\bar{3}^{2}_{001}\|\tau_{(0.0,0.0,0.0)}\}$
7	$\begin{bmatrix}-1.0 & 0.0 & 0.0\\0.0 & -1.0 & 0.0\\0.0 & 0.0 & -1.0\end{bmatrix}$	$\begin{bmatrix}0.0 & 1.0 & 0.0\\1.0 & 0.0 & 0.0\\0.0 & 0.0 & 1.0\end{bmatrix}$	$\begin{bmatrix}0.0\\0.0\\0.0\end{bmatrix}$	$\{\bar{1}\|\|m_{1\bar{1}0}\|\tau_{(0.0,0.0,0.0)}\}$
8	$\begin{bmatrix}-1.0 & 0.0 & 0.0\\0.0 & -1.0 & 0.0\\0.0 & 0.0 & -1.0\end{bmatrix}$	$\begin{bmatrix}1.0 & -1.0 & 0.0\\0.0 & -1.0 & 0.0\\0.0 & 0.0 & 1.0\end{bmatrix}$	$\begin{bmatrix}0.0\\0.0\\0.0\end{bmatrix}$	$\{\bar{1}\|\|m_{120}\|\tau_{(0.0,0.0,0.0)}\}$
9	$\begin{bmatrix}-1.0 & 0.0 & 0.0\\0.0 & -1.0 & 0.0\\0.0 & 0.0 & -1.0\end{bmatrix}$	$\begin{bmatrix}-1.0 & 0.0 & 0.0\\-1.0 & 1.0 & 0.0\\0.0 & 0.0 & 1.0\end{bmatrix}$	$\begin{bmatrix}0.0\\0.0\\0.0\end{bmatrix}$	$\{\bar{1}\|\|m_{210}\|\tau_{(0.0,0.0,0.0)}\}$
10	$\begin{bmatrix}-1.0 & 0.0 & 0.0\\0.0 & -1.0 & 0.0\\0.0 & 0.0 & -1.0\end{bmatrix}$	$\begin{bmatrix}0.0 & -1.0 & 0.0\\-1.0 & 0.0 & 0.0\\0.0 & 0.0 & -1.0\end{bmatrix}$	$\begin{bmatrix}0.0\\0.0\\0.0\end{bmatrix}$	$\{\bar{1}\|\|2_{1\bar{1}0}\|\tau_{(0.0,0.0,0.0)}\}$

Table S1. (continue) Elements of the spin space group for G-type AM phase.

11	$\begin{bmatrix}-1.0 & 0.0 & 0.0\\ 0.0 & -1.0 & 0.0\\ 0.0 & 0.0 & -1.0\end{bmatrix}$	$\begin{bmatrix}1.0 & 0.0 & 0.0\\ 1.0 & -1.0 & 0.0\\ 0.0 & 0.0 & -1.0\end{bmatrix}$	$\begin{bmatrix}0.0\\ 0.0\\ 0.0\end{bmatrix}$	$\{\bar{1}\|\|2_{210}\|\tau_{(0.0,0.0,0.0)}\}$
12	$\begin{bmatrix}-1.0 & 0.0 & 0.0\\ 0.0 & -1.0 & 0.0\\ 0.0 & 0.0 & -1.0\end{bmatrix}$	$\begin{bmatrix}-1.0 & 1.0 & 0.0\\ 0.0 & 1.0 & 0.0\\ 0.0 & 0.0 & -1.0\end{bmatrix}$	$\begin{bmatrix}0.0\\ 0.0\\ 0.0\end{bmatrix}$	$\{\bar{1}\|\|2_{120}\|\tau_{(0.0,0.0,0.0)}\}$

VIII. Symmetry Analysis for Non-relativistic Spin Splitting

Following the pseudoscalar theory of spin, the symmetry operations for the altermagnetic (AM) $CoPSe_3$ can be described as

$$\{\mathcal{E}, \mathcal{P}, 2\mathcal{C}_3, 2\mathcal{S}_6\} + \mathcal{R}_S\{3\mathcal{M}_d, 3\mathcal{C}_2\}. \qquad (S-3)$$

Here, $\mathcal{R}_S$ is an antisymmetric operation that reverses the signs of spin and magnetic moments without affecting the wave vector k. **Figure S10** gives spin-degenerate k-paths under symmetry operations $\mathcal{R}_S\mathcal{M}_d$ and $\mathcal{R}_S\mathcal{C}_2$.

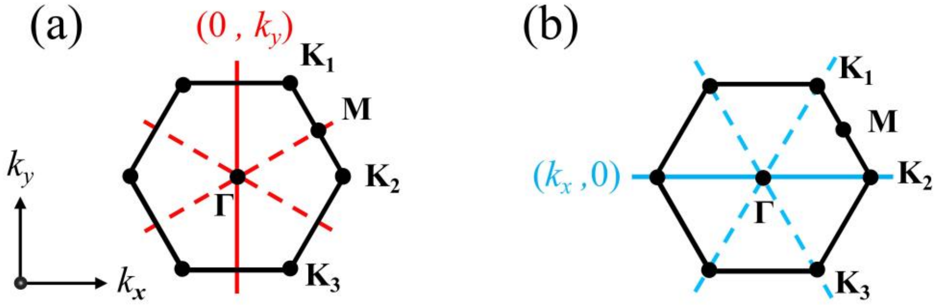

Figure S10. Spin-degenerate k-paths under symmetry operations a) $\mathcal{R}_S\mathcal{M}_d$ and b) $\mathcal{R}_S\mathcal{C}_2$.

IX. $\mathcal{PT}$-symmetry Exclusion of BCD in NAHE

Both the quantum metric and the Berry curvature dipole (BCD) can contribute to the nonlinear anomalous Hall effect (NAHE), but their contributions are governed by distinct symmetry requirements. The BCD is expressed as:[8-10]s

$$\mathrm{D}_{\alpha\beta}(\boldsymbol{k}) = -\sum_{n}\int \frac{d^2k}{(2\pi)^2}\frac{\partial \varepsilon_{\boldsymbol{k}}^{n}}{\partial k_\beta}\Omega_{\alpha}^{n}(\boldsymbol{k})\frac{\partial f_{kn}}{\partial \varepsilon_{\boldsymbol{k}}^{n}}, \qquad (\mathrm{S}-4)$$

where $\Omega_{n\boldsymbol{k}}^{\alpha}$ is the band-projected Berry curvature, and $\partial\varepsilon_{\boldsymbol{k}}^{n}/\partial k_\beta$ is the corresponding group velocity. The behavior of this integrand under various symmetry operations determines whether the BCD can yield a nonzero contribution to the NAHE.

Under the time-reversal ($\mathcal{T}$) symmetry, we have

$$\mathcal{T}\Omega_{n}^{\alpha}(-\boldsymbol{k}) = -\Omega_{n}^{\alpha}(\boldsymbol{k}), \qquad (\mathrm{S}-5)$$

$$\mathcal{T}\frac{\partial \varepsilon_{\boldsymbol{k}}^{n}}{\partial(-k_\beta)} = -\frac{\partial \varepsilon_{\boldsymbol{k}}^{n}}{\partial k_\beta}. \qquad (\mathrm{S}-6)$$

Accordingly, the integrand $\Omega_{n\boldsymbol{k}}^{\alpha}\partial\varepsilon_{\boldsymbol{k}}^{n}/\partial k_\beta$ is $\mathcal{T}$-even, allowing BCD to exist in $\mathcal{T}$-symmetric materials.

Under spatial inversion ($\mathcal{P}$) symmetry, we have

$$\mathcal{P}\Omega_{n}^{\alpha}(-\boldsymbol{k}) = \Omega_{n}^{\alpha}(\boldsymbol{k}), \qquad (\mathrm{S}-7)$$

$$\mathcal{P}\frac{\partial \varepsilon_{\boldsymbol{k}}^{n}}{\partial(-k_\beta)} = -\frac{\partial \varepsilon_{\boldsymbol{k}}^{n}}{\partial k_\beta}. \qquad (\mathrm{S}-8)$$

The product $\Omega_{n\boldsymbol{k}}^{\alpha}\partial\varepsilon_{\boldsymbol{k}}^{n}/\partial k_\beta$ becomes odd under $\mathcal{P}$. As a result, its integration over the whole Brillouin zone cancels, leading to a vanishing BCD contribution in $\mathcal{P}$-symmetric systems.

Under the combined $\mathcal{PT}$-symmetry operation, the Berry curvature acquires an overall sign change while the group velocity remains unchanged. This means the product $\Omega_{n\boldsymbol{k}}^{\alpha}\partial\varepsilon_{\boldsymbol{k}}^{n}/\partial k_\beta$ transforms as an odd function of $\boldsymbol{k}$. Upon integration over the full Brillouin zone, this odd function cancels out, thereby prohibiting any BCD-driven contribution to the NAHE in $\mathcal{PT}$-symmetric systems. This conclusion also aligns with previous studies.[11-12] Given these considerations, in our $\mathcal{PT}$-symmetric C-type AFM $CoPSe_3$ bilayer, the NAHE is indeed dominated by the quantum metric, while the BCD contribution is explicitly excluded by symmetry.

X. Anisotropy of the AHE and the NAHE

In **Tables S2**, **S3**, and **S4**, we list the corresponding magnetic point group (MPG) and indicate the presence (√) or absence (×) of the AHE and the NAHE in both $CoPSe_3$ monolayer and bilayer, respectively. Note that the spin orientations for in-plane cases (*xy*-plane) of different magnetic phases are labeled as $\hat{n}(\varphi)$, when the out-of-pane (along the *z*-axis) case is described separately.

Table S2. The MPGs and the presence or absence of AHE and NAHE for Néel-type AFM $CoPSe_3$ monolayer.

Monolayer	**Néel-type AFM** Néel vectors $\hat{n}(\varphi)$	MPGs	Linear AHE	Second-order NAHE
state1	$\hat{n}(0°)$	$2'/m$	×	√
state2	$\hat{n}(90°)$	$2'/m$	×	√
state3	$\hat{n}(135°)$	$2/m'$	×	√
state4	out-of-plane	$\bar{3}'m$	×	×

Table S3. The MPGs and the presence or absence of AHE and NAHE for C-type AFM $CoPSe_3$ bilayer.

Bilayer	**C-type AFM** Néel vectors $\hat{n}(\varphi)$	MPGs	Linear AHE	Second-order NAHE
state1	$\hat{n}(0°)$	$2'm$	×	√
state2	$\hat{n}(90°)$	$2'm$	×	√
state3	$\hat{n}(135°)$	$2/m'$	×	√
state4	out-of-plane	$\bar{3}'m$	×	×

Table S4. The MPGs and the presence or absence of AHE and NAHE for G-type AM $CoPSe_3$ bilayer.

Bilayer	**G-type AM** Néel vectors $\hat{n}(\varphi)$	MPGs	Linear AHE	Second-order NAHE
state1	$\hat{n}(0°)$	$2/m.1$	×	×
state2	$\hat{n}(90°)$	$2/m.1$	×	×
state3	$\hat{n}(135°)$	$2'/m'$	√	×
state4	out-of-plane	$\bar{3}m.1$	×	×

It is clear that the linear AHE exists exclusively in the AM phase and exhibits a pronounced anisotropy. In AA-stacked $CoPSe_3$ bilayers, the presence or absence of linear AHE is closely related to the symmetry breaking induced by the magnetic arrangement and the resulting nonrelativistic spin splitting. Thus, the existence of linear AHE depends on the orientation of Néel vector. Consequently, control of the linear AHE can be effectively achieved through combined manipulation of both crystal symmetry and magnetic configuration. For NAHE, anisotropy is observed in both monolayers and bilayers. Due to the symmetry restriction, both AHE and NAHE are prohibited with out-of-plane Néel vectors.

XI. Spin Splitting in Trilayer System

The structure of the AA-stacked $CoPSe_3$ trilayer is illustrated in **Figures S11a** and **S11b**. The top view of $CoPSe_3$ trilayer resembles that of the bilayer while the side view is similar to bilayer for the same stacking pattern. Through the symmetry analysis of three different interlayer magnetic configurations presented in **Figure S11c**, we find that both the $\mathcal{PT}$-symmetry in the C-type AFM and the G-type AFM configurations are preserved, while the $\mathcal{PT}$-symmetry is broken in the hybrid magnetic configuration.

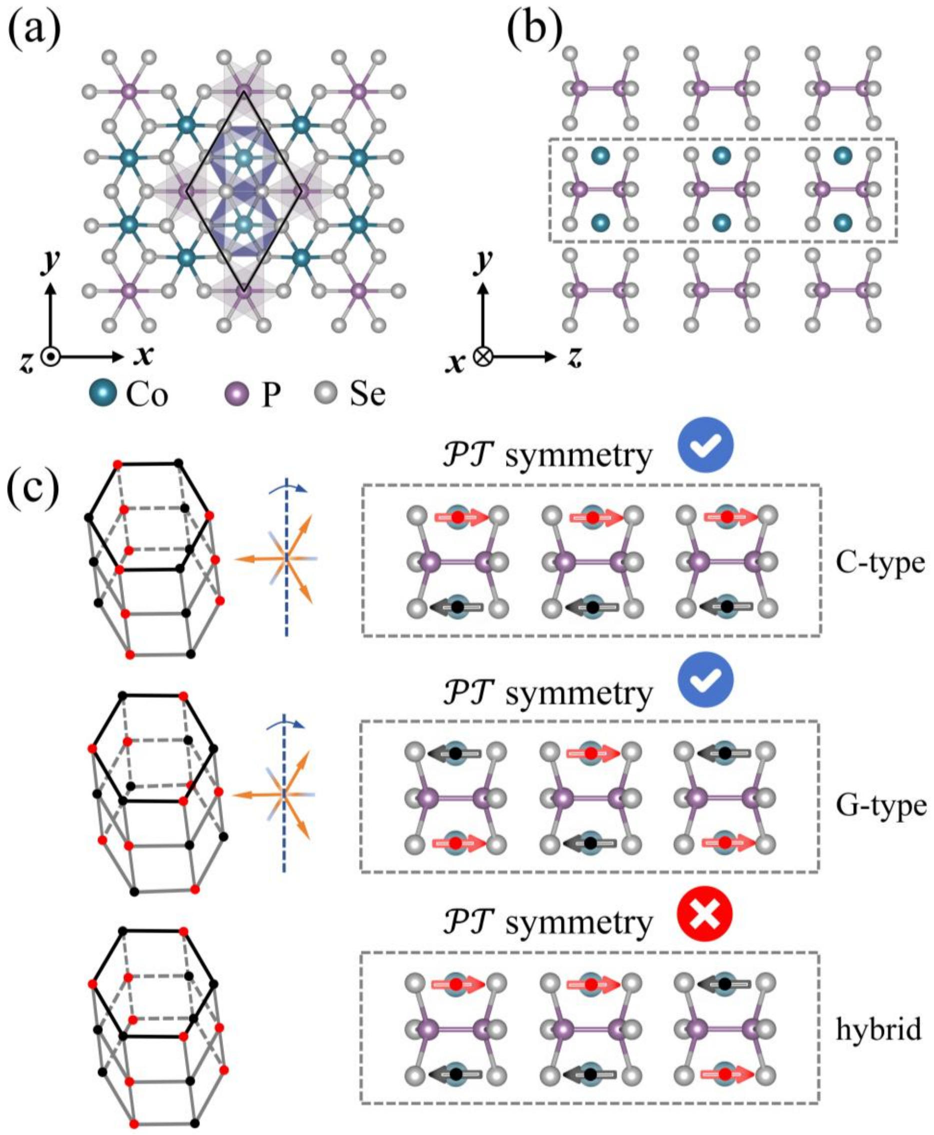

Figure S11. Crystal and magnetic configurations of $CoPSe_3$ trilayer. a) Top and b) side views of $CoPSe_3$ trilayers, with the primitive unit cell outlined by solid black diamonds. c) Illustration of spin-dependent $\mathcal{PT}$ symmetry in trilayer, where the red and blue points/arrows indicate the magnetic moments of Co ions with opposite spin orientations.

To further verify the electronic structure, we show the band structures of the three magnetic configurations corresponding to the generic *k*-paths in **Figure S12**. Obviously, nonrelativistic spin splitting occurs in the symmetry-broken interlayer hybrid magnetic states. These findings highlight the potential occurrence of nonrelativistic spin splitting in multilayer hexagonal systems.

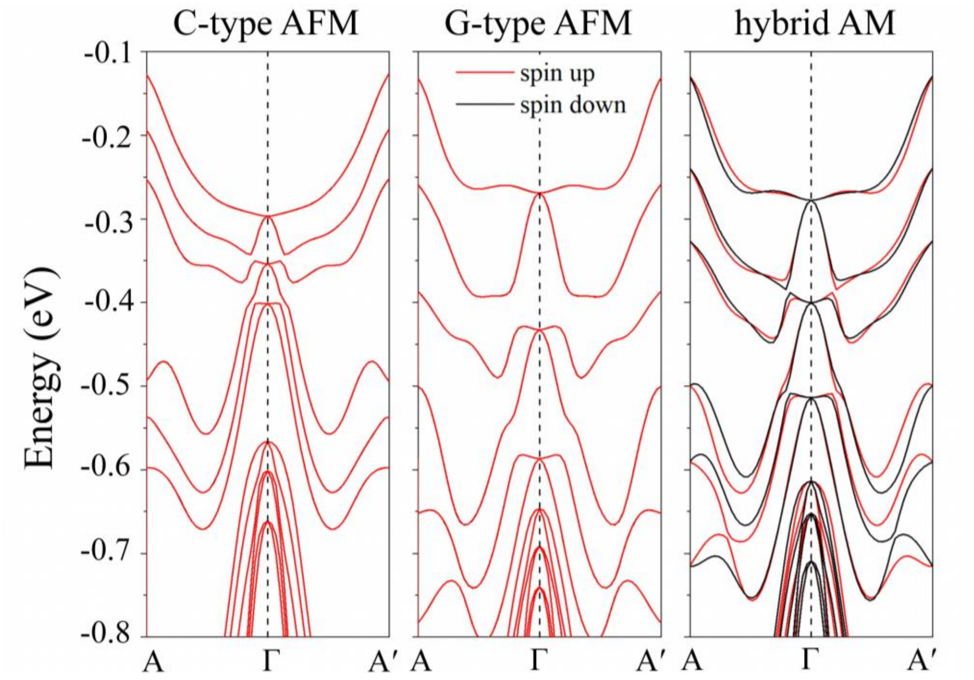

Figure S12. Band structures of $CoPSe_3$ trilayer with different magnetic configurations.

XII. References

[1] H. J. Xiang, E. J. Kan, S.-H. Wei, M. H. Whangbo, X. G. Gong, Predicting the spin-lattice order of frustrated systems from first principles, *Phys. Rev. B* **2011**, *84*, 224429.

[2] Z. Sun, X. Li, H. Jin, Z. Zhao, Y. Wei, J. Wang, Magnetic influence on phonon frequencies in two-dimensional FeOCl: Insights from spin-phonon coupling, *Phys. Rev. B* **2024**, *110*, 165415.

[3] Y. Xu, A. Ray, Y. T. Shao, S. W. Jiang, K. Lee, D. Weber, J. E. Goldberger, K. Watanabe, T. Taniguchi, D. A. Muller, K. F. Mak, J. Shan, Coexisting ferromagnetic-antiferromagnetic state in twisted bilayer CrI_3, *Nat. Nanotechnol.* **2022**, *17*, 143.

[4] X. L. Yang, X. Y. Xie, W. Q. Yang, X. H. Wang, M. L. Li, F. W. Zheng, Stacking-dependent interlayer magnetic interactions in $CrSe_2$, *Nanotechnology* **2024**, *35*.

[5] P. D. Lomenzo, L. Collins, R. Ganser, B. H. Xu, R. Guido, A. Gruverman, A. Kersch, T. Mikolajick, U. Schroeder, Discovery of Nanoscale Electric Field-Induced Phase Transitions in ZrO_2, *Adv. Funct. Mater.* **2023**, *33*.

[6] H. C. Ma, X. M. Chen, Y. F. Han, J. Zhang, K. Q. Wen, S. Y. Cheng, Q. Y. Zhao, Y. J. Wang, J. Y. Wu, J. Y. Shao, Ice-Enabled Transfer of Graphene on Copper Substrates Enhanced by Electric Field and Cu_2O, *Adv Sci* **2024**, *11*.

[7] B. I. Weintrub, Y. L. Hsieh, S. Kovalchuk, J. N. Kirchhof, K. Greben, K. Bolotin, Generating intense electric fields in 2D materials by dual ionic gating, *Nat. Commun.* **2022**, *13*.

[8] Z. Z. Du, C. M. Wang, H.-Z. Lu, X. C. Xie, Band Signatures for Strong Nonlinear Hall Effect in Bilayer WTe_2, *Phys. Rev. Lett.* **2018**, *121*, 266601.

[9] I. Sodemann, L. Fu, Quantum Nonlinear Hall Effect Induced by Berry Curvature Dipole in Time-Reversal Invariant Materials, *Phys. Rev. Lett.* **2015**, *115*, 216806.

[10] D. Xiao, M. C. Chang, Q. Niu, Berry phase effects on electronic properties, *Rev. Mod. Phys.* **2010**, *82*, 1959.

[11] C. Wang, Y. Gao, D. Xiao, Intrinsic Nonlinear Hall Effect in Antiferromagnetic Tetragonal CuMnAs, *Phys. Rev. Lett.* **2021**, *127*, 277201.

[12] H. Liu, J. Zhao, Y. X. Huang, W. Wu, X. L. Sheng, C. Xiao, S. A. Yang, Intrinsic Second-Order Anomalous Hall Effect and Its Application in Compensated Antiferromagnets, *Phys. Rev. Lett.* **2021**, *127*, 277202.
